# The therapeutic potential of inorganic polyphosphate: A versatile physiological polymer to control coronavirus disease (COVID-19)

**DOI:** 10.7150/thno.59535

**Published:** 2021-04-15

**Authors:** Hadrian Schepler, Xiaohong Wang, Meik Neufurth, Shunfeng Wang, Heinz C. Schröder, Werner E. G. Müller

**Affiliations:** 1 Department of Dermatology, University Clinic Mainz, Langenbeckstr. 1, 55131 Mainz, Germany.; 2 ERC Advanced Investigator Grant Research Group at the Institute for Physiological Chemistry, University Medical Center of the Johannes Gutenberg University, 55128 Mainz, Germany.

**Keywords:** polyphosphate, nanoparticles, SARS-CoV-2, viral receptor-binding domain

## Abstract

**Rationale:** The pandemic caused by the novel coronavirus SARS-CoV-2 is advancing rapidly. In particular, the number of severe courses of the disease is still dramatically high. An efficient drug therapy that helps to improve significantly the fatal combination of damages in the airway epithelia, in the extensive pulmonary microvascularization and finally multiorgan failure, is missing. The physiological, inorganic polymer, polyphosphate (polyP) is a molecule which could prevent the initial phase of the virus life cycle, the attachment of the virus to the target cells, and improve the epithelial integrity as well as the mucus barrier.

**Results:** Surprisingly, polyP matches perfectly with the cationic groove on the RBD. Subsequent binding studies disclosed that polyP, with a physiological chain length of 40 phosphate residues, abolishes the binding propensity of the RBD to the ACE2 receptor. In addition to this first mode of action of polyP, this polymer causes in epithelial cells an increased gene expression of the major mucins in the airways, of *MUC5AC* and *MUC1*, as well as a subsequent glycoprotein production. MUC5AC forms a gel-like mucus layer trapping inhaled particles which are then transported out of the airways, while MUC1 constitutes the periciliary liquid layer and supports ciliary beating. As a third mode of action, polyP undergoes enzymatic hydrolysis of the anhydride bonds in the airway system by alkaline phosphatase, releasing metabolic energy.

**Conclusions:** This review summarizes the state of the art of the biotherapeutic potential of the polymer polyP and the findings from basic research and outlines future biomedical applications.

## Introduction

Until the beginning of the 19^th^ century airborne infections, caused by bacterial or viral respiratory pathogens, were viewed as uncommon or ephemeral phenomena. This estimation changed diametrically in the early 20^th^ century with the emergence of influenza which peaked pandemically in 1918 (Spanish flu; 20 to 50 million death toll), 1957 (Asian flu; 1 to 4 million deaths worldwide), and 1968 (Hong Kong flu; 1 to 4 million people killed globally). At present a new pandemic is spreading, the coronavirus disease 2019 (COVID-19), which is caused by the respiratory syndrome coronavirus 2 (SARS-CoV-2), now reaching over 2 million deaths in January 2021 1. Estimates showed that the basic reproductive rate (R_0_) of 2.5 (range 1.8-3.6) in the SARS-CoV-2 pandemic is similar to that of SARS-CoV and the 1918 influenza pandemic, but higher than in the MERS-CoV pandemic with 0.9 2. The inherent property of all viruses, including SARS-CoV-2 3,4, is to mutate, a dynamic that can lead to even more aggressive SARS-CoV-2 variants with distinct focal dissemination pattern(s).

It is surprising that SARS-CoV-2 is causing a wide range of human symptoms ranging from acute self-resolving cough, fever, loss of sense of smell or taste, to severe illness and death. Certainly the viral load against which the individuals are exposed to through the air as well as the viral load in the organism of the patients, which affects the various organs, determine the severity of the disease 5. Here two strategies are available that can prevent the onset and the development of the disease; firstly the natural, innate immune system and secondly the adaptive immune protection caused by vaccination. In the present review it is highlighted that it is a physiological polymer in the body that gives individuals the beneficial property to resist against viral infection. It is polyphosphate (polyP). This inorganic, physiological polymer can prevent infection in its earliest phase, at the step of attachment of the virus to the target cells. As such polyP is a member of the innate immunity protection. The preclinical studies are completed, animal studies have been performed - the clinical trial appears to be recommended.

In general, the threat posed by airborne infectious pathogens cannot be fully assessed. The lasting damage to the population and society remains largely unknown. The difficulties in sampling the viruses from the air as well as their low concentration in this environment, do not allow a prescient estimate. Assessing the risk of viruses in body fluids such as blood (human immunodeficiency virus [HIV]), urine (measles and mumps) or cells (human polyomavirus) seems to be easier than that of airborne viruses that are transmitted, inhaled or exhaled, to infect mucosal dendritic cells in the nasopharynx 6. While for influenza transmission via fluid drops requires close contact, aerosol particles are most likely the vehicle through which SARS-CoV-2 is transmitted 7.

Fortunately, the progress in the natural sciences has helped considerably to discover the etiologic agents of airborne infections since 1546, when Fracastoro postulated for tuberculosis “seminaria contagiorum” as a transmissible agent 8. It was a heroic achievement to uncover the secret of HIV within about 2 years 9 and now within 12 months the basic biology of SARS-CoV-2 10. It is especially the molecular biology that drives the molecular virology. Nevertheless, the epidemiological projections remain empiric and are not yet based on causal-analytical models, as there are still open questions about the life cycle of the pandemic caused by SARS-CoV-2.

## The origin and transmission route of SARS-CoV-2

### Origin of SARS-CoV-2

The location for the first outbreak of COVID-19 with its etiologic agent SARS-CoV-2 was identified around a local seafood market in Wuhan-China in December 2019 11. Subsequent sequencing of the RNA genome provided evidence that the closest phylogenetic relatives of the human sequences are two bat SARS-related coronaviruses, RaTG13 and RmYN02, with a sequence identity of 96.2% 12. Coronaviruse(s) with higher sequence identity may exist but have not yet been detected or spread. In any case, it can be assumed that SARS-CoV-2 has been transmitted from animals to humans *via* a zoonotic process. Based on phylogenetic analyses, it is presently assumed that SARS-CoV-2 probably also diverged from a bat-derived ancestor between 1948 and 1982 13.

### Transmission route of SARS-CoV-2: Respiratory tract

Like other coronaviruses, SARS-CoV-2 is dispersed and most likely transmitted directly or by aerosols or droplets *via* the respiratory tract 14. Aerosols have a size of < 5 μm, while airborne droplets can reach sizes of up to 500 μm. In addition, other routes like fecal-oral or indirect transmission *via* fomites could likewise contribute to the global dissemination of the virus. Highest viral load has been identified in throat swabs at the symptom onset, and infectiousness also peaked at the onset of the symptoms 14. Importantly, long-distance airborne dispersal through central ventilation systems that are remote from patient areas is also possible, since it turned out that exhaust filters from a station were positive for SARS-CoV-2 15.

**Figure 1:**
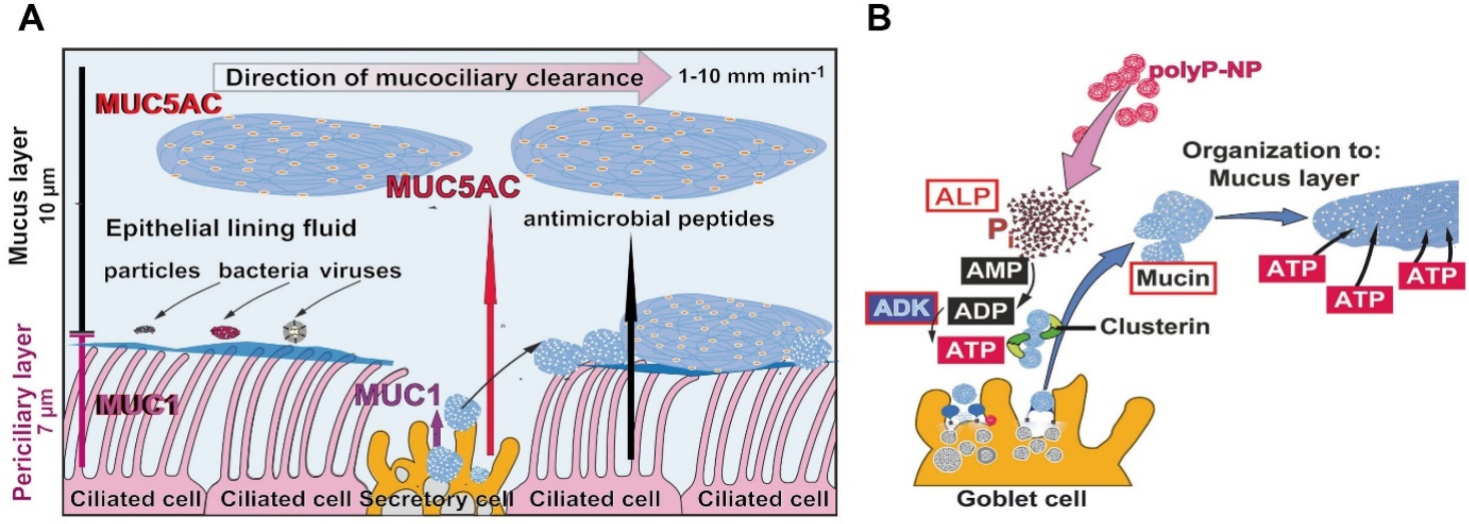
The mucus barrier protecting against COVID-19 infection. (**A**) Schematic outline of the airway epithelium. The major cell types are the secretory cells (goblet cells), which produce the mucins, and the ciliated cells. MUC1 is a membrane-associated mucin that remains in the periciliary layer and enwraps the surface cilia protruding from the ciliated cells. The second major mucin, MUC5AC, forms the epithelial lining fluid which integrates foreign particles, bacteria and viruses. The ciliated cells drive the mucus, mainly composed of MUC5AC, out of the airway system. The velocity of the mucociliary clearance is fast with 1 to 10 mm min^-1^. Besides of the mucins antimicrobial peptides fight against the microbes. (**B**) Sketch of the formation and organization of the mucus layer from individual mucin molecules through ADP/ATP supported by the HSP-like clusterin. Polyphosphate (polyP) packed in nanoparticles (polyP-NP) acts as an extracellular cargo of metabolic energy. In the presence of the enzyme alkaline phosphatase (ALP) the phosphoanhydride bonds in polyP are cleaved and the released Gibbs free energy is transferred to AMP under formation of ADP. Subsequently, ADP generates ATP and AMP catalyzed through the adenylate kinase (ADK).

Application of molecular testing methods showed that SARS-CoV-2 first infect the nasopharynx and then spread to the lower respiratory tract 16. It is important that the highest expression of viral genes among the cells of the respiratory system is measured in the nasal epithelial layer and there in clusters of goblet cells and to a smaller extent also in ciliated cells 17. Subsequent detailed immunohistochemical analyses revealed that the goblet cells and the ciliated cells as well as the club cells are highly infected, but the basal cells are not 18. Most of the normal human airway is lined by a pseudostratified epithelium, a characteristic columnar epithelium formed of a close to single layer of cells. A basement membrane-like barrier with pores exists through which immune cells can migrate 19. The basal cells are multipotent stem cells that are lined at the basis of the epithelium and act as precursor cells, from which ciliated, secretory, and goblet cells differentiate during repair processes 20. The cells that produce the aqueous secretion that covers the mucous epithelia are the goblet cells, which are relatively abundant, while the ciliated cells, which comprise 50 to 80% of the epithelium, use their cilia to drive foreign materials in the respiratory tract towards the mouth, from where they can be swallowed or expectorated 21. The length of each cilium is about 7 μm 22; Figure 1A.

### Additional routes of transmission: olfactory mucosa and corneal epithelium

In addition to the airway system, SARS-CoV-2 can enter the circulation system via the nasal olfactory mucosal and/or corneal epithelium route. Both epithelia are also covered by a mucus layer composed of MUC1 and MUC5AC as the major constituents 23,24. In addition, the receptor for the attachment of the virus to the host cells, the angiotensin-converting enzyme 2 (ACE2), exists in both tissues; in the olfactory neuroepithelium 25 and also in the ocular surface tissue 26.

Focusing on the nasal mucosal epithelium it has been proven that the influenza A viruses replicate not only in the upper respiratory tract but also in the nasal respiratory epithelium. Recently, the latter route has been considered as the major driver for the viral transmission via the air 27. Therefore, the public has been advised to take the necessary precautions and to prevent person-to-person transmission 28. This relates also to medical/nursing staff in the clinics. The entry of SARS-CoV-2 to the nervous system by crossing the neural-mucosal interface of the olfactory mucosa is well established 28. This route allows the virus to contact the olfactory mucosal, endothelial and nervous tissue and finally the olfactory as well as the sensory nerve endings.

With respect to a spreading through conjunctival contact with SARS-CoV-2 patients it has been determined that the viral RNA is also present in the mucus covering the cornea 29. Genes encoding ACE2 and other viral S-protein modifying enzymes and molecules have been identified 30. The tear film is rich in antimicrobial substances, especially in lactoferrin and α-lysine, while lysozyme is absent 31. The antiviral activity is restricted to lactoferrin only. During the 1918 influenza pandemic a transmission *via* the eye has been proposed 32, but this view is considered premature 33. At present, it appears unlikely that a spread of SARS-CoV-2 via the ocular tear film occurs 33.

Alveolar macrophages, phagocytes, are most likely the first sensors of the respiratory tree that link SARS-CoV-2 with the immune system 34. In this way, this cell type is a key player controlling the balance between defending against pathogens and tolerating the invaders. The macrophages reside in the pulmonary alveoli, near the pneumocytes, but are separated from the wall 35. While alveolar macrophages are also involved in the removal of dust particles, they eliminate microorganisms from the respiratory surfaces. Although these macrophages are poor presenters of antigens to T-lymphocytes 36 they transport those antigens to the lung-draining lymph nodes which are close to them 37. Therefore, the alveolar macrophages are not only the first sentinel of the respiratory system for SARS-CoV-2 infection but also the first and major early responder to respiratory virus infections 34. After evading the early innate immunity response, these cells release interferon, parallel to an infiltration of the immune cells that circulate in the blood system and cause a release of pro-inflammatory cytokines. The subsequent stimulation of Th1/Th17 cells by viral epitopes can cause the aggravated inflammatory responses, the “cytokine storms”. These immunopathological events are often associated with pulmonary edema or pneumonia. Cytotoxic T lymphocytes are recruited to kill virus-infected cells in the lungs. In parallel, B cells/plasma cells are attracted and activated to produce antibodies that are specific to SARS-CoV-2. The aim is to inactivate the viruses in the airway system and to confer a systemic immunity in selected organs 38.

Based on the severe acute respiratory syndrome (SARS) of the 2003 epidemic, the mean time to seroconversion was estimated to be 14 to 20 days after infection and to reach a maximum after 19 days 39. The virus is spread around the body via the circulating immune cells which engulf the pathogen. By infecting the epithelial cells of the trachea, bronchi, bronchioles and lungs, the virus also invades non-immune cells and causes organ dysfunction, including gastrointestinal symptoms, abnormal liver function and splenic atrophy. In turn, a parallelism is seen between SARS-CoV-2 and HIV; both viruses then slowly destroy subsets of lymphocytes in addition to the target cells. However, the SARS virus is more aggressive. It infects and destroys immune cells much faster and in a more disastrous fashion 40.

At present, there is no doubt that wearing face masks, cloth masks or surgical masks, is an effective solution to control the COVID-19 pandemic 41,42. They are efficient enough to reduce the exposure to aerosol-laden SARS-CoV-2 by more than 50%. With the introduction of 3D printing technology, these protective devices can be fabricated in a personalized manner and effectively 43,44. They shall be worn also by pathologists inspecting potentially infectious corpse 45. The additively manufactured masks as medical devices are especially advisable also for the fabrication of Ambu bag ventilators 46.

## Mucus, the infection barrier for SARS-CoV-2

Often neglected is the fact that SARS-CoV-2 can only hardly reach the cells of the epithelia directly because the viral particles have to penetrate the bulky mucus layer 47. The respiratory epithelium is overlayed with a 1 to 10 µm thick mucus blanket 22,48; Figure 1A. The velocity of the mucus that is transported on the respiratory epithelium is fast, with 1 to 10 mm min-1 48,49. In turn, the mucociliary clearance and transit time is 12 to 15 min under physiological conditions 50.

The mucus layer lining the epithelium is structured. The mucus-secreting goblet cells of the tracheobronchial airway are interspersed in the epithelium and are connected with the adjacent cells, the goblet cells, by tight junctions, allowing them to act as a selective barrier. This histological organization implies that the mucus-secreting cells, the goblet cells, and 60% of the ciliated cells, line the surface of the epithelium 21. In turn, the surface of the epithelium is separated from the lumen of the airway canals firstly by the 7 µm long cilia with MUC1 and secondly by the 10 µm airway mucus gel, consisting of MUC5AC, which forms the epithelial lining fluid that is located on top of MUC1 and the cells with their radiating cilia (Figure 1A).

ATP is the driving metabolite for mucin secretion and interacts with the P2Y2 receptors at the apical part of the goblet cells 51. This process is controlled separately from the mucin production 52. The coordinated and continuous production of ATP maintains the secretory machinery and the continuous flow of mucins to establish the protective mucin barrier 22. The discharge of the mucins appears as an explosive exocytic release from the mucin grana after sensitization of the cells through ADP or ATP 53. During this burst the volume of the mucus increases by 1,000-fold.

Compared to other extracellular compartments such as the mesenchyme, the connective tissue or the cartilage, in which the fibrous filamentous and non-filamentous macromolecules are arranged in a highly organized manner, a precise ordering must also be predicted for the mucus layer. There the individual mucin molecules must be organized into flexible, homogeneous layers. Even more, the mucus in the mucous barrier must allow the filaments and the protruding cell-membrane integrated heparan sulfate macromolecules to be embedded. To organize the arrangement of the macromolecules in the extracellular airway mucus metabolic energy is required. Further down it is outlined that polyP acts as ATP generator in the extracellular space 54. Predominantly heat shock proteins (HSP) control intracellularly the correct folding of macromolecules, processes that require ATP as energy donor. In the mucous epithelial cells the major HSPs, HSP27, HSP72 and HSP73, are present 55. Recently it has been proposed that in the mucin layer clusterin might act as a HSP-folding protein 56. Clusterin is provided with an ADP binding motif 57, which might indicate that this molecule functions as an HSP. This protein has been identified in the sputum 58, indicating that clusterin may be involved in airway inflammation. In Figure 1B it is sketched how the organization of the mucus layer could be supported by polyP.

The family of mucins is grouped into over 20 different glycoproteins with two defining characteristics 22. They are primarily highly O-glycosylated and frequently composed of tandem repeats of amino acids along their protein backbone. From there, the so-called mucin domain, the glycosylation side groups extend. The mucins, abbreviated MUC, are divided into two subfamilies, the secreted mucins and the membrane-tethered mucins. The prominent mucin of the group of the secreted mucins is the polyanionic mucin MUC5AC (size of ~650 kDa) which is dominant in the mucus of the airway system (Figure 1A). It is layered on top of the periciliary layer and acts as a support for the fluid and mobile phase of the mucus, which is used to remove foreign particles, bacteria and viruses during the mucociliary clearance. Most of the mucins comprise within their polymeric chains, at both ends, intermolecular disulfide bonds allowing the formation of bulky homomultimeric assemblies. These gel-forming glycoproteins of the respiratory tract epithelia protect the mucosa from bacterial and viral infections as well as from chemical damage by binding to the inhaled organic or inorganic microorganisms or particles that are subsequently removed by the mucociliary fluid transport system.

The second group the membrane-tethered mucins are membrane-spanning molecules that function as cell surface receptors. The major form is MUC1 in the airway mucus. They are smaller than the secreted mucins with around 300 kDa 59. MUC1 shows cell adhesive properties and can act as both an adhesive and an anti-adhesive protein. Due to its trans-membranous structure the molecule is involved in cell signaling via reversible phosphorylation processes and protein-protein interactions. Among them are the ERK, SRC and NF-κ-B pathways.

The mucin molecules are nm-small, globular molecules with a hydrodynamic diameter of 10 nm for MUC1 60; the size of MUC5AC is 4-5 nm in diameter 61. These dimensions are extremely small in comparison to MUC1 or MUC5AC in the periciliary layer or in the mucus layer, respectively. The mucins are arranged in a multi-scale hierarchical way as structured fluids 62 that require an energy-dependent hydration transformation on the surface of epithelial airway system, into which the purinoceptors of the apical membrane are integrated 63. The homeostasis, the degree of mucus hydration in the epithelial airway cultures, is sensed through an interaction between the cilia and the overlying mucus layer 64,65. Data indicate that this process, the mucus hydration in the airway system, is controlled by ATP present in the extracellular space 63,66. This view is supported by results showing that inhibition of ecto-ATPase restores the physiological hydration of the airway surfaces in cystic fibrosis 67. Later in this review it is discussed that polyP can be considered as a generator for ATP synthesis in the extracellular space 54.

The mucus layer is a multi-functional hydrogel which provides both a physical and a chemical barrier against microbial infections. As a hydrogel the mucus is highly elastic, flexible and rheologically changeable and has the property to break apart and reanneal again to a protective barrier 68. The mucus can efficiently entrap pathogens and also inactivate them to non-infectious particles. The anti-pathogenic properties of the mucus is strengthened by the presence of the antibacterial and antiviral ingredients 69-71.

In the literature conflicting results exist about the size of the particles which can penetrate the mucus layer. Originally it has been proposed that particles, viruses or antibodies can freely diffuse within milliseconds through the mucus if they are smaller than the mesh size of the mucins 72. Later it was found that particles with a larger size (≥500 nm) are also transported through the mucus, even though with a slower migration rate. The transport is strongly dependent on the physical properties of the mucus and the particles 73.

The influenza virus can be trapped within the human airway mucus without binding to sialic acid units on the mucins chains 74. Similarly, neutralizing antibodies can also pass through the mucus mesh network 75. In humans immunoglobulin A, the major secretory immunoglobulin in the mucosa, contributes to ~70% of the body's total Ig production. This fraction is abundantly present in the upper airways. These antibodies are effective and their level correlates with the resistance against the influenza virus infection 76. Antibodies have also been raised against human SARS-CoV-2 and tested *in vivo*, in mice. The animals were exposed to a high titer of SARS-CoV-2 pseudovirus 77. The material was applied by both nasal and lung spraying. After treatment the animals were effectively protected. The same application has been successfully tested against H5N1 influenza virus in mice 78.

## The mucus layer in COVID-19 patients

It is often not sufficiently taken into account that SARS-CoV-2 does not directly reach the cells of the epithelia, but has to penetrate the bulky mucus layer 47. Recently some data on SARS-CoV-2 infection have been published in this regard 79. It is reported that the gel-forming mucins MUC5AC and MUC5B, the primary structural components of the fluid layer on top of the periciliary cells of the mucosal epithelium, as well as the transmembrane mucins of the respiratory tract, MUC1, MUC4, and MUC16, undergo differential glycosylation after inflammation and exposure to proinflammatory cytokines and microbial ligands 47.

Also in COVID-19 patients, dry cough is an important natural defense mechanism of the respiratory tract and taken as a typical symptom of the disease 80. This feature is ambiguous in view of reports showing that in COVID-19 patients an increased production of MUC1 and also MUC5AC occurs 81. In this report it is discussed that the increased level of MUC species results in a high viscosity of the mucus in the small airways 82. Interesting is the finding that during the progression of COVID-19 infection a damage of the cilia of the ciliated cells takes place. These cells promote the spreading of the virus to the deeper lung parenchyma 83. Especially the cells in the airway epithelium are dependent on the supply of ATP required for the synchrony of the ciliary movement under *in vitro* conditions 84. In this system an optimal concentration of ATP of 10^-5^ to 10^-3^ M is determined. These values exceed the concentration measured in the mucus *in vivo* 85. The published data indicate that the ciliated epithelial cells in the airway system are the prime target for SARS-CoV-2, while the secretory cells are only occasionally infected.

## Attachment of the virus to the host cell surface

The life cycle of SARS-CoV-2 is dissected into six phases; virus entry, translation of viral replication machinery, replication, translation of viral structure proteins, virion assembly, release of virus 86. On all these levels potential antiviral drug candidates have been published. The present study focuses on the first phase in the replication cycle of the virus, after which the virus can enter the host organism and its cells. Therefore, a blockade of this attachment step prevents viral infection. The attachment process of the virus to the target cell is complex and involves both membrane fusion and entry *via* clathrin-mediated endocytosis 87. The main components of the virus involved are the viral receptor binding domain (RBD) at the distal end of the viral spike protein and the cellular ACE2 receptor. This crucial interaction between the RBD and ACE2 is affected by polyP; here is the focus of the review.

In addition, a further mechanisms has been introduced, the engulfment of the virus particles in nanodecoys that are mimicking cell membranes. Such a mechanism was first demonstrated with Zika viruses and then generalized as a trapping way for COVID-19 and finally also for other infectious diseases 88-90. Such an interaction between nanodecoys and pathogen complexes is not only advantageous for the inactivation of the virus particles, but also for the development of safe vaccines.

### The interaction of the viral spike with the cellular ACE2 receptor

**Figure 2:**
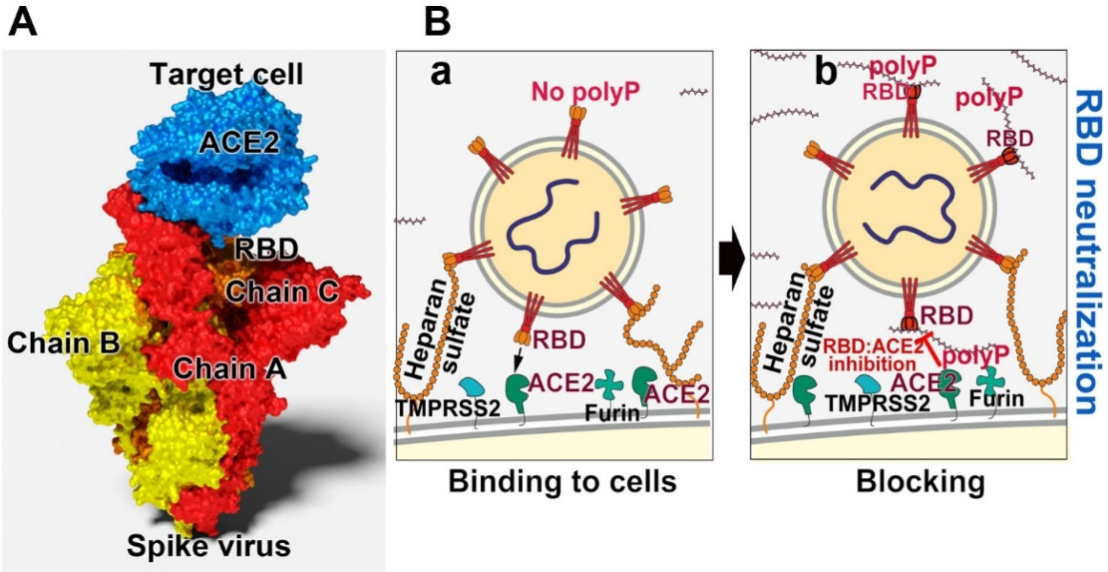
Blocking the binding of SARS-CoV-2 to the ACE2 receptor of the host. (**A**) Scheme of the spike (S)-protein of the virus. The spike is composed of three chains onto which the RBD is attached. The RBD interacts with the ACE2 receptor on the target cell. (**B**) Effect of polyP on the interaction of the virus with the target cell. (**a**) This process is mediated by the viral RBD and the cellular ACE2 receptor. Associated with this complex are the two enzymes furin and TMPRSS2, which cleave the spike prior to virus entry into the cells. (**b**) Both polyP and heparan sulfate have binding sites for the RBD. It has been shown by our group that polyP binds strongly to the viral RBD and causes a blockade of the interaction with the cells.

The size of the genome of Coronaviridae is large with 25 to 32 kb [kilobases] of RNA. Therefore the Coronaviridae represent the viruses with the largest known RNA. The size of the RNA of the RNA virus HIV-1 is much smaller with less than 10 kb 91. The SARS-CoV-2 virion has a globular morphology with a diameter of 118 to 136 nm and 9 to 12 nm large surface projections formed by the viral spike (S) glycoproteins 92; Figure 2A. These proteins extend from the virus envelope, in which a viroporin is embedded as a further envelope protein 93. The number of spikes amounts to 100 to 200 per virion. The virus particles cannot interact directly with the surface of the airway epithelial cells, but must first penetrate through a bulky mucus sheath with mucin molecules. In addition they are hindered by a rim of membrane associated heparan sulfate molecules (~50 nm) which are tightly anchored in the cell membrane 94. The latter molecules even interact with ionic bonds with the virus particles 95. Finally, the virions have the 7 μm long cilia as a hurdle that they have to overcome in order to reach the airway epithelium.

The ACE2 molecule, which is associated with the host cell surface, acts as the receptor for the virions to attach to the host cells 96; Figure 2B. There the entry of the particles into the cells occurs via two mechanisms, through an endosomal pathway or by plasma membrane fusion 97. The binding partner of ACE2 is the spike (S)-protein, which protrudes from the virus body. This molecule is a trimer of about 180 kDa, chain A, B and C, and each of the monomers is composed of two subunits, S1 and S2; on the distal terminus of the units a RBD is attached 98,99; Figure 2A. After binding of the virus to the plasma membrane, to the ACE2 receptor, the fusion of the viral envelope with the host cell membrane takes place 100. During the binding process the S-protein becomes proteolytically cleaved between the S1 and the S2 subunits through the TMPRSS2 serine protease 96,101; Figure 2B. Alternatively but less important, entry of the virus into the cells follows a cathepsin L-dependent endo-lysosomal uptake of the virions into the target cell 102. The heparan sulfate molecule tends to bind to the RBD 103.

The expression studies disclosed that the highest density of ACE2 is on the two nasal epithelial cells, the goblet cells and the ciliated cells, suggesting that this entry route is prevalent for the virus particles 17. After infection these cells undergo pyroptosis (lytic programmed cell death) and release damage-associated molecular patterns and also ATP 104. These events cause the release of pro-inflammatory cytokines and chemokines in the neighboring epithelial cells and endothelial cells as well as in alveolar macrophages. The mediators attract monocytes, macrophages and T-lymphocytes, which accumulate at the site of infection and promote inflammation. During this process, the integrity of the epithelial layer is disrupted, and in addition to pyroptosis, vascular leakage occurs. The latter process is also seen in SARS-CoV-2 patients 105. As a result, macrophages, T-lymphocytes and monocytes can infiltrate into the mucus.

### The distinctive feature of the viral RBD: the cationic groove

Any drug that attempts to inhibit a viral infection or the subsequent viral replication needs to interfere with a viral molecule that is crucially and specifically involved in the viral life cycle. Most often enzymes are the drug targets, like genome replicating enzymes, polymerases, or enzymes that activate a pro-drug to a functionally active drug. Examples are inhibitors of DNA polymerases, like disclosed for the DNA-dependent DNA polymerase in herpes simplex virus type 1 106 with adenine arabinoside (9-β-d-arabinofuranosyladenine) 107. A further alternative is to prevent the association of a viral non-enzymatic molecule with its corresponding cellular receptor, like the prevention of a virus attachment molecule with its cellular receptor. An example is sulphoevernan a polyanionic polysaccharide which inhibits the HIV viral life cycle during the attachment of the virus to the target cells 108. It is indicative that the same principle can also be applied for the interaction of the S-protein of SARS-CoV-2 with the cellular ACE2 receptor.

**Figure 3:**
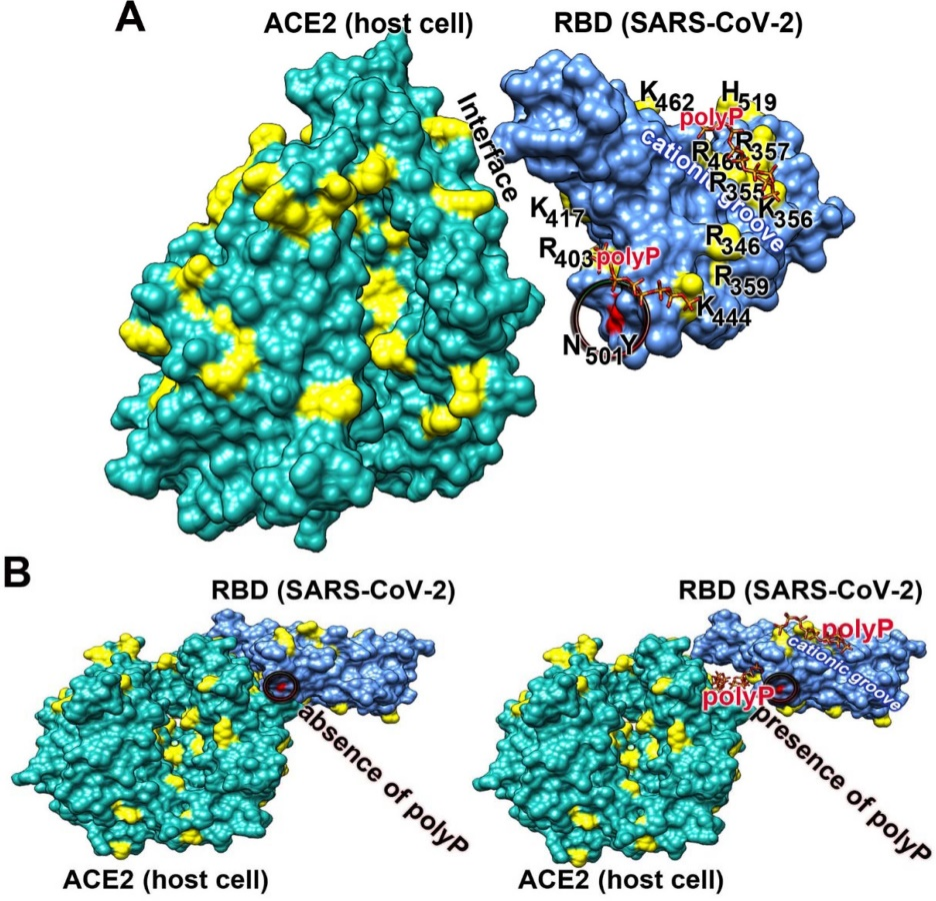
Clustering of basic amino acids in the cationic groove of the SARS-CoV-2 RBD and interaction with polyP. (**A**) Basic amino acids (aa; in yellow) on the surface of the RBD and ACE2. In the cationic groove of the RBD the basic aa are arranged in a continuum. The newly developed mutant in the UK, SARS-CoV-2 B.1.1.7, comprises an exchange of asparagine (N) by tyrosine (Y) at position 501 in the RBD sequence. This mutation (circled) has no binding site to polyP. (**B**) The polymer polyP strongly binds to the cationic groove and also to basic aa at the interface between ACE2 and the RBD. This steric hindrance prevents the docking between the two components.

In a comprehensive study it has been shown that primarily polyanionic or poly-zwitterionic compounds inhibit the binding of viruses to their cellular receptors 109. Focusing on SARS-CoV-2 it has been determined that the viral S-protein is slightly positively charged, while the corresponding cellular receptor ACE2 is, under physiological conditions, negatively charged 110. The sensor of SARS-CoV-2 for its cellular receptor is the RBD, which is located at the distal terminus of the S-protein (Figure 3A). It has an amino acid (aa) length of 218 aa with an isoelectric point of 8.9 111. The sequence of the RBD is highly conserved within the pathogenic human *Coronaviridae* subfamilies. The expect value of 2e^-166^ is remarkably small, meaning that the mutation pressure on the RBD is most likely low resulting in a low mutation rate 112. Very indicative is the presence of an unusually organized aa pattern on the surface of the RBD, the cationic groove. This groove is built by a stretch of basic aa of the RBD, a protein which is characterized by a surplus of basic aa (with Arg [arginine] + Lys [lysine] + His [histidine]: 11 + 10 + 1) over the presence of the acidic aa (Asp [aspartate] + Glu [glutamate]: 15 113,114); Figure 3A-B. In this cationic groove, the basic aa are arranged in a clustered pattern, formed from aa which are spaced 3.8 and 5 Å between their aa units. Interestingly enough Arg-rich peptides are frequently found in viral cell-penetrating peptides like in the HIV-1 Tat protein which binds to the hair-looped structure of the viral TAR RNA 115. This viral Tat peptide likewise shows a stretch of six Arg, two Lys and only one polar/not charged aa (Gln) and functions during penetration of the peptide into cells 116,117. The electrostatic forces coming from the basic aa are intensified by adjacent acidic epitopes of the acidic aa aspartic acid and glutamic acid 118. Almost perfectly matching with the basic aa of the cationic groove are the individual phosphate units of the physiological polyP polymer, which are linked together via acid anhydride linkages 54. The spacing between the phosphate units, which are tightly associated with the surface of the viral RBD, is ~3 Å.

Here it is also stressed that the new mutant of SARS-CoV-2, B.1.1.7, which emerged in the UK, comprises an exchange of the asparagine (N) by tyrosine (Y) at aa position 501 of the RBD sequence 119; Figure 3A. This exchange is not linked with the cationic groove and has therefore no influence on the polyP binding propensity to the RBD.

## What is polyphosphate?

The only physiological inorganic polymer that has been conserved throughout the evolution of living systems is polyP 120.

### The biological inorganic polyanion

**Figure 4:**
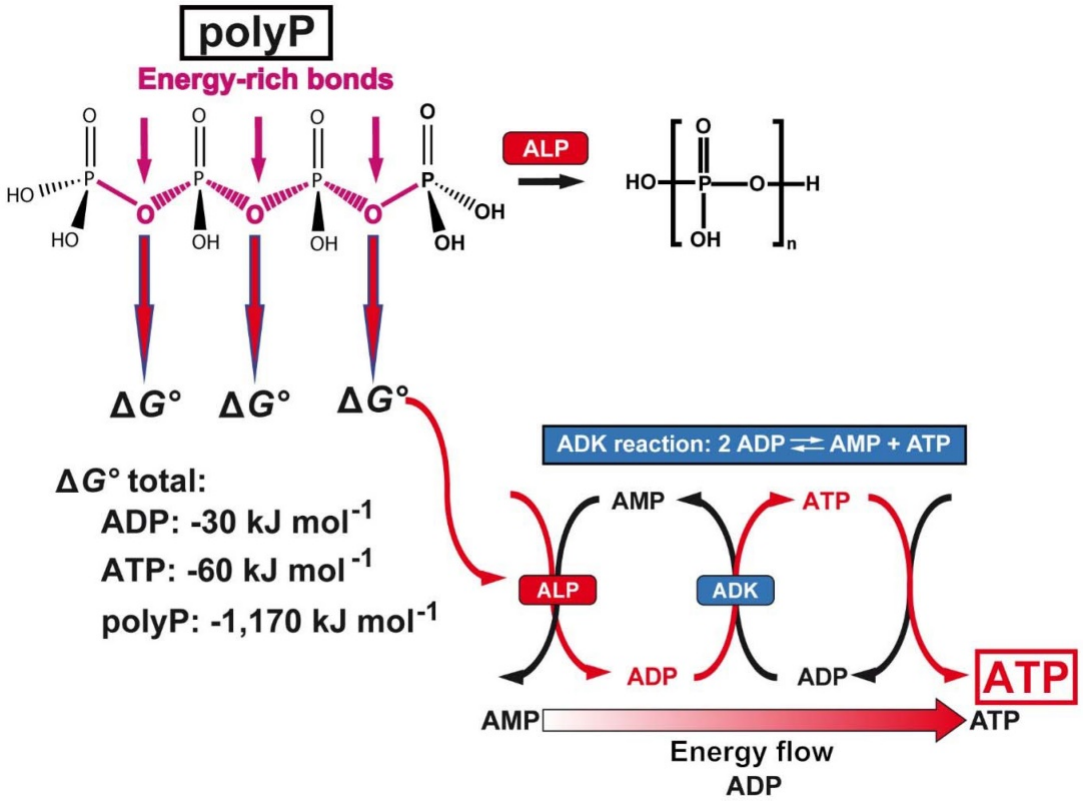
PolyP a cargo and ATP generator in the extracellular space. Above: Structure of polyP and hydrolytic cleavage of the terminal phosphate by ALP. The free energy, Gibbs free energy (ΔG°), that is released during cleavage of the energy-rich acid anhydride linkages of polyP with a chain length of 40 P_i_ units, compared to ADP or ATP, is listed below. In addition, the reaction scheme for the transfer of the energy from the phosphoanhydride bonds of polyP to ADP and finally ATP *via* the ALP and ADK is shown.

Inorganic polyphosphate (polyP) is abundantly present in every cell, prokaryotic or eukaryotic, and there as an amorphous linear polymer composed of repeated phosphate [PO_4_^3-^] units, which are linked together by tens to hundreds of high energetic phosphoanhydride bonds 121; Figure 4. This polymer is intracellularly synthesized from ADP/ATP, two metabolites discovered by Lohmann 122. Later on, Kulaev, Sylvia Ruth and Arthur Kornberg pushed ahead the discoveries on polyP (reviewed in: 123). Two intracellular organelles exist that are rich in polyP, the mitochondria and the acidocalcisomes 124,125. ATP, generated during the oxidative phosphorylation in the mitochondria, is released into the cytoplasm through the voltage-dependent anion channels of the outer mitochondrial membrane. Subsequently, ATP reaches the acidocalcisomes, 50 to 300 nm large cell organelles which are ubiquitously present from bacteria to animal cells. These organelles are rich in polyP (150 mM), Ca^2+^ (2 M) and ATP (400 mM); 125. polyP is synthesized by the vacuolar transporter chaperone (Vtc) complex in the membrane of the acidocalcisomes 126. Especially in the mast cells and the blood platelets polyP is synthesized and partially encapsulated in the acidocalcisomes as well 127. In mast cells polyP has a long half-life of ~40 d 128, while it is shorter in platelets with about 8 to 9 d 129. However, the capacity of the platelets to produce polyP is much higher. From the megakaryocytes (50-100 μm in diameter) which intensively synthesize polyP, the polymer is passed during their differentiation to the platelets; around 2000 to 5000 new platelets are released from one megakaryocyte 130. Importantly, two fractions of polyP are produced in the acidocalcisomes; first polyP with a chain length of 70-75 Pi units 131,132 and second a polyP pool with longer chain lengths of 200 to 1000 Pi units 133. These two fractions are released in two different states, as soluble shorter chain polymers and as longer chain molecules 134. Importantly, the short-chain polyP is released from the platelets and remains in a soluble form, while the long-chain polyP is encapsulated into 100 to 200 nm large particles and then released from the platelets. In the blood polyP, both the short-chain and the long-chain fraction, undergoes gradual enzymatic hydrolysis, catalyzed by the alkaline phosphatase (ALP) 135,136.

### PolyP undergoes coacervation

At neutral pH and in the presence of divalent cations, like Ca^2+^ or Mg^2+^, polyP forms a coacervate. This reflects a phase separation process during which the colloids associate together and form an aqueous gel-like phase 137. The process is driven by an increase of the zeta (ζ) potential of the particles; the lower the potential the higher is the propensity for coacervate formation. The reduction of the ζ potential is achieved by the presence of peptides or proteins and increases the biocompatibility of the respective polymer.

### Effect of polyP on the blood clotting system

In the circulating blood the concentration of polyP, after full platelet activation, is 0.5 to 3 μg mL^-1^ 124,138. Again, the soluble short-chain polyP exists at a size of ∼50 P_i_ units, and the longer polymer (∼250 units) that is encapsulated into nanoparticles becomes associated with the surface of the platelets 133. In an earlier report polyP has been attributed to accelerate the blood clotting process, primarily by binding to the factor XII within the clotting cascade 132. In continuation it has been proposed that polyP drives an initiation of proinflammatory and pro-coagulant disorders. In later detailed studies it has been demonstrated that any effect of polyP on blood clotting is dependent on the length of the polymer 139. Under physiological conditions and in the presence of the physiologically circulating polyP with a chain length of 50 Pi units polyP has no effect on the blood clotting 140,141. Finally, it has been demonstrated that polyP even inhibits the blood clotting cascade by lowering the Ca^2+^ and the thromboxane A_2_ during platelet aggregation 142.

### Generation of extracellular ATP

PolyP has two major functions in humans. It provides, with phosphate, the raw material for the formation of the bone hydroxyapatite 143-145. The *in vitro* as well as *in vivo* data disclosed that polyP functions as a regeneratively active polymer if applied as biomimetic amorphous nanoparticles prepared from short-middle size polyP (chain length of ~40 P_i_ units); 146. It is important that the application form of polyP is amorphous and not crystalline since any inorganic deposit in the human body relies on amorphous seeds, prior to the transformation to a crystalline phase 147. This fabrication process copies the reactions running *in vivo*; meaning that the particle formation takes place at a stoichiometric surplus of divalent cations like Ca^2+^ or Mg^2+^, conditions which are also found in the acidocalcisomes 125. Crucially important during preparation of the particles is the adjustment of the pH to the alkaline direction 146. In nature, the pH in the acidocalcisomes also controls the polyP deposition, which proceeds in an alkaline environment 148.

The second unique feature is that the phosphate units of the polyP chain are linked with each other *via* energy-rich phosphoanhydride bonds (Figure 4). Since the studies of Lipmann 149 it has been generally accepted that any amount free energy that is generated in a living system during catabolic metabolic reactions is - at least in part - stored in energy-rich compounds such as ATP, which is then available for subsequent metabolic processes. The energy released from polyP exceeds by far the one which can be obtained from ATP or ADP; the Gibbs free energy (ΔG°) for ATP (total amount of possible energy released) is -60 kJ mol^-1^, for ADP -30 kJ mol^-1^ and for polyP with a chain length of 40 P_i_ units, -1,170 kJ mol^-1^. Only a part of the energy is dissipated as heat. In order to recover the energy trapped in the phosphoanhydride bonds, the polymer must undergo enzymatic cleavage. The enzyme in eukaryotes mediating this process is the alkaline phosphatase 135 which hydrolyses the polymer in a processive way 54. ADP is generated in this first step, which then serves as a substrate for the adenylate kinase (ADK) to generate ATP 150,151. During this transfer of P_i_ units from polyP to AMP (formation of ADP; ALP reaction) and from ADP to ATP (with simultaneous formation of AMP; ADK reaction), the energy stored in polyP is transferred to ATP. Both reactions can be blocked by inhibitors, ALP by levamisole and ADK by Ap_5_A 152.

The two enzymes ALP and ADK are present in the airway mucus and also control the homoeostasis of ATP there 153-155.

## Inhibition of the interaction of viral RBD and cellular ACE2 by polyP

At the beginning it was very conspicuous that a distinctly delimited area exists on the surface of the RBD that asked for a function. With the dynamic modeling approach, we were able to show that polyP fits perfectly on this cationic groove. Subsequently, we have shown that this polymer sensitively binds to the RBD and causes its inactivation 113,114.

### The interaction RBD with ACE2

**Figure 5:**
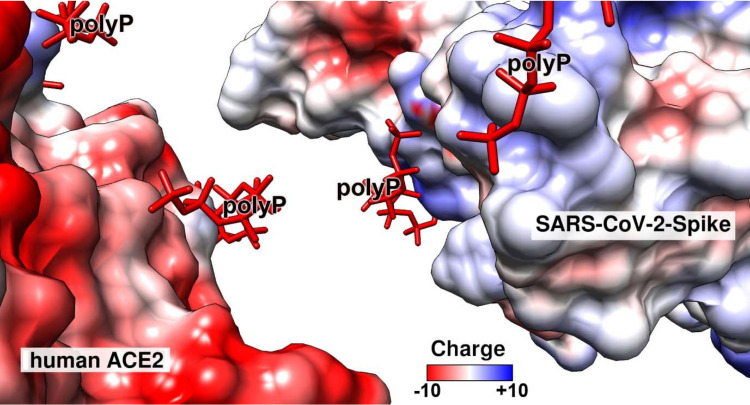
The electric charge distribution on the surface of the human ACE2 receptor (left) and the RBD, located on the distal terminus of the S-protein of SARS-CoV-2 (right), based on the charges of the respective aa residues and following Coulomb's law. The color field marks the range from -10 (overall negative charge) to +10 (positive charge). The regions where the highly negatively charged (in red) polyP polymers bind to the two partner molecules are in blue (positively charged aa residues). The molecular models for ACE2 and the SARS-CoV-2 RBD were taken from PDB-ID 6M0J, while the polyP molecule was extracted from PDB-ID 5llf.

The RBD domain of the viral S-protein tightly binds to the ACE2 receptor of the target cells. The interaction is strong with a dissociation constant KD of ~120 nM, as assessed by the kinetic on-rate from single-molecule force spectroscopy experiments 156. In parallel the KD value for the RBD: ACE2 interaction was determined by the sensitive surface plasmon resonance analysis to be 14.7 nM 157. The difference of these numbers is attributed to the two different methods used, but the high binding strength is reflected in these two contributions. The thermodynamic properties of the ACE2 receptor also indicates that binding of the RBD to ACE2 is the only major interacting system between the virus and the cells. Surely, under physiological conditions the strength of binding of these two molecular partners is even stronger due to multivalent electrostatic interactions. The conclusion from these findings is that sliding of an anionic polymer such as polyP into the interface of ACE2 and the RBD would reduce or even block this interaction (Figure 5). This view is strengthened by the fact that the cationic groove on the surface of the SARS-CoV-2 RBD exactly matches the polyanionic sequence of the polyP 113,114; Figure 5. Such an organized surface patterning primarily formed by the clustered Arg aa units on the surface of the RBD, which is even associated with and flanked by Asp and Glu, establishes and amplifies a strong intramolecular proton transfer 118. Those reactive centers facilitate a quasi-covalent reaction/addition of the guanidinium group of Arg to the phosphate units in a polyP chain.

The strength of the electrostatic interaction of two molecules is determined by electrostatic and electrical forces and is high if the binding partners are oppositely charged 158,159. In turn, the strength of interaction between the two partner molecules, ACE2 and RBD, was calculated based on the electric charge distribution onto the two molecules (Figure 5). The calculation revealed that the interaction of the polyanionic polyP can be predicted over the positively charged regions that are mainly built from the basic Arg residues. The polyP has also the necessary steric prerequisites to flexibly bind to the surface of the proteins, since it has rotational flexibility at the anhydride linkages of 130° and 102° 54. This property also allows the proteins to be enwrapped by polyP and facilitates interaction with the surfaces of the molecules at a close distance of ∼3 Å.

### Modification of Arg at RBD

The Arg units on protein surfaces can be provided with a stronger electrostatic interacting potential by adjacent acidic aa units 118. In addition, Arg has the potential to interact via its guanidinium group with non-polar aromatic and aliphatic side chains above and below the guanidinium plane 160. In order to mimic those transformations, the Arg units in the RBD were modified with 1,2-cyclohexanedione (CHD) in a borate buffer, to allow the modified Arg aa, DHCH-Arg, to protrude through the hydrate shell around the protein molecule 161,162.

### Inhibition of RBD : ACE2 interaction

The strength of inhibition by polyP of the interaction between the SARS-CoV-2 RBD and the target cell ACE2 has been determined by applying a highly sensitive ELISA-like binding assay. The recombinant cellular ACE2 is bound to the solid phase and the RBD, labeled with biotin, remains in the fluid phase. The RBD with its cationic groove is exposed to the test compound polyP and then added to the ACE2 113,114. The quantity of the complex formed between RBD and ACE2 is determined using a chemiluminescence based method.

**Figure 6:**
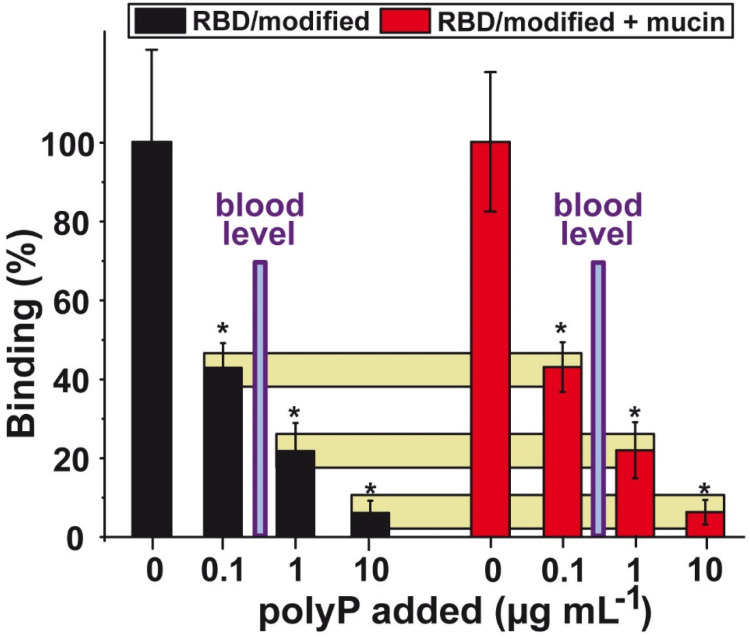
Effect of polyP on the binding affinity between the RBD and ACE2. For this series the Arg residues on the surface had been modified with CHD to meet physiological conditions. Left columns (in black) indicate the interaction in the absence of mucin and the right ones (in red) show the effect of mucin in the binding assay (100 µg mL^-1^) on the interaction. The yellow horizontal bars connect corresponding inhibition values; absence or presence of mucin. Adapted with permission from ref. Neufurth et al. ref. 114. Copyright 2019, MDPI, Basel, Switzerland.

The Arg residues of the RBD were treated with CHD in order to imitate the most likely physiological conditions, an increased electron density distribution around the Arg (DHCH-Arg). A significant reduction of the binding strength was measured at a concentration of 0.1 µg mL^-1^ of Na-polyP, with 58%. Higher concentrations of 1 µg mL^-1^ and 10 µg mL^-1^ of Na-polyP caused a reduction by 78% and 94%, respectively (Figure 6).

To highlight, the concentration of the soluble polyP in the circulating blood is in the range of 0.5 to 3 μg mL^-1^. In turn, this finding implies that under physiological conditions the level of polyP in the blood should be high enough to effectively block the RBD : ACE2 interaction.

### Interaction with mucin

**Figure 7:**
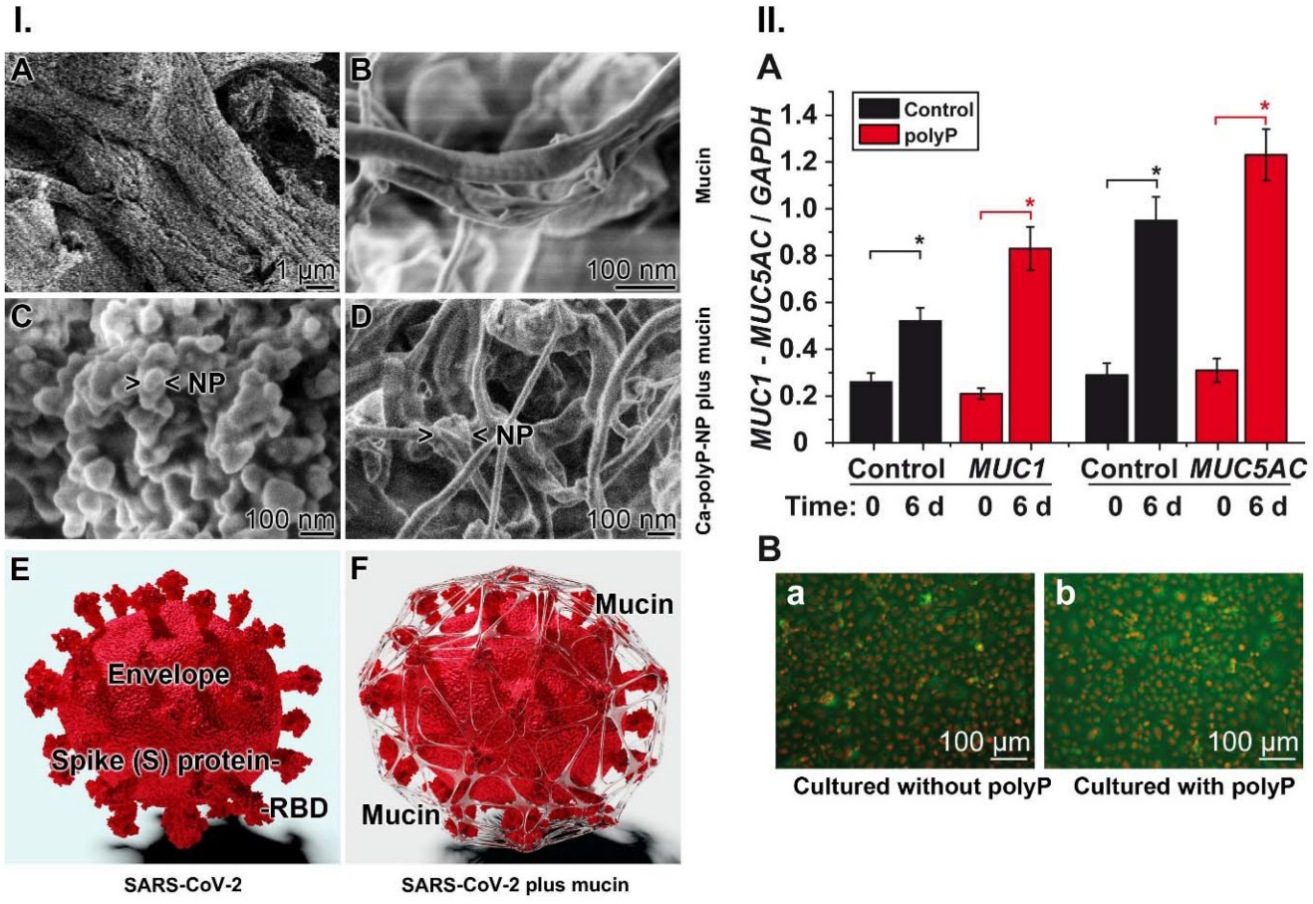
Interaction of Ca-polyP-NP with mucin and effect of polyP on mucin expression. (**I.-A** and **I.-B**) Fibrous mucin in the absence of polyP at different magnifications; SEM. (**I.-C**) Ca-polyP-NP in the absence of Na-polyP. (**I.-D**) The nanoparticles were added to a mucin solution of 100 µg mL^-1^, enriched with 10 mM Ca^2+^ like under physiological conditions. Most of the nanoparticles (NP) are integrated into the coacervate. (**I.-E** and **I.-F**) Wrapping of SARS-CoV-2 by the mucin-polyP coacervate; a cartoon. The 3D model was created by the Visual & Medical Arts Unit and the Electron Microscopy Unit, Research Technologies Branch, Rocky Mountain Labs, NIAID (2020). (**II.-A**) Effect of 10 μg mL^-1^ of Na-polyP on the steady-state-expression of the genes encoding for MUC1 and MUC5AC (red bars); in the controls (black bars) no polyP was added. The A549 cells were incubated for 6 d. Then the gene expression level was determined by qRT-PCR with cDNA for either *MUC1* or *MUC5AC*. (**II.-B**) Increase of the mucin protein level in A549 cells in the absence [collagen-matrix alone; control] (**a**) or presence (**b**) of polyP [collagen-matrix supplemented with 100 µg mL^-1^ of polyP]. MUC1 protein was detected in the cells by immunofluorescence using human MUC1 antibodies. The mucin areas flash in green.

Under physiological *in vivo* conditions the virus particles cannot reach directly the epithelial cells of the airways. It is the ~20 µm thick mucus layer which prevents an immediate interaction. The dominant organic components of the mucus layer are the mucins. They are a family of high molecular weight glycoproteins that, in the presence of an aqueous environment, form a mucin gel that has a fibrous structure. The diameter of the mucin fibers is around 50 nm 163-165; Figure 7.I.A-B.

In order to conduct an application study of polyP to determine the potential protective effect of the polymer on the airway epithelia, a formulation was prepared that contains both soluble Na-polyP and polyP packed in amorphous nanoparticles (Ca-polyP-NP). The diameter of the untreated nanoparticles is ~100 nm (Figure 7.I.C). If mucin is exposed to a 10:1 mixture of Na-polyP and Ca-polyP-NP in PBS, enriched with 10 mM Ca^2+^ in order to mimic physiological conditions 166, the polyP NP (Figure 7.I.C) undergo coacervation. After the incubation time of 12 h some nanoparticles can still be seen that are attached to the mucin scaffold via the coacervate (Figure 7.I.D).

The presence of mucin in the polyP fraction added to the RBD : ACE2 interacting system does not cause a significant change of the observed polyP-caused inhibition. In this series of experiments mucin (concentration of 100 µg mL^-1^) was added to polyP 47; Figure 6. The data show that polyP, both in the absence or the presence of mucin, strongly reduces the attachment of the viral RBD to the cellular receptor. This blockade is reached at physiological conditions 47,114.

The effect of polyP on the binding affinity of the RBD is long-lasting. The binding of polyP to the RBD is not reduced after repetitious washing steps. The presence of mucin also does not impair the binding of the polymer. In turn, one could predict that the dissociation constant for polyP is even lower than the K_D_ value of the RBD to the cellular ACE2 receptor, which is in the range of ~ 1-100 nM 167. A cartoon about the binding of mucin-polyP coacervate on the surface of the SARS-CoV-2 is given in Figure 7.I.E-F.

### Induction of mucin synthesis by polyP

PolyP not only acts as a scaffold for the mucus layer on the surface of the airways, but also causes an increase in the production of mucin in human alveolar basal epithelial A549 cells 47. Based on the finding that polyP acts as a regeneratively acting material 144 and additionally produces ATP in the extracellular space 54 it was promising to determine if polyP causes also an increased expression of mucin in those cells. Mucus production is known to require a substantial amount of metabolic energy 168.

For the gene expression study in the human cell line A549, the steady-state-expression levels of MUC1 and MUC5AC were determined, since these mucin genes encode the major mucin species. MUC1 is the predominant tethered mucin in the human airways, and MUC5AC is the major gel-forming mucin in this system 169. The cells were incubated in the absence or presence of 10 µg mL^-1^ of polyP for 6 d 47. In the qRT-PCR studies an increased expression was measured also in the controls during incubation; the MUC1 level increased by 2-fold and the MUC5AC level increased by 3-fold. However, in the presence of polyP a significant enhancement of the expression levels was found, by 2.9-fold for MUC1 and 3.2-fold for MUC5AC (Figure 7.II.A). In order to verify if the higher expression can also be observed on the protein level mucin was determined by immunofluorescence analysis using an antibody raised against human MUC1 (Figure 7.II.B). The images show that the intensity of the mucin-antibody, detected by a green fluorescent antibody, is obviously stronger in the polyP-treated cells (Figure 7.II.B-a) compared to control (Figure 7.II.B-b). This finding underscores that the gene expression level in cells treated with polyP is higher (Figure 7.II.A), which is also reflected in an increased protein synthesis in these A549 cells (Figure 7.II.B-b versus the control in Figure 7.II.B-a).

### Druggable characteristics of the COVID-19 disease: From respiratory transmission to pathogenesis - the molecular triad

SARS-CoV-2 is entering the target individual via the nasal cavity and/or the upper respiratory tract. It is the distinctive feature of COVID-19 that the majority of persons infected with SARS-CoV-2 has a mild infection of the upper respiratory tract, while some people suffer of severe infections of the acinar airways, which cause disastrous, fatal pneumonia 170. This meta-analysis disclosed that SARS-CoV-2 RNA shedding can continue in respiratory and stool samples for a longer period. Importantly, the duration of virus shedding in the upper respiratory tract is longer (~17 days), while this period in the lower respiratory tract is short at around 14 days. The maximum has been determined in the upper respiratory tract at 83 days, and in the lower tract with 60 days, and 126 days in stool samples and 60 days in serum. An apparent discrepancy exists between the presence of live virus (no viable virus was detectable beyond day 9 of illness) and persistently high viral loads 170. These findings reflect that the virus SARS-CoV-2 titers peak in the upper respiratory tract during the first week of illness. In contrast to SARS-CoV-2 infection, which spreads rapidly, the clinical features of SARS-CoV and MERS-CoV show a slower spreading 171.

The existing data forcibly show that the viral load, the quantity of virus, in the upper respiratory tract remains high, in an area where SARS-CoV-2 has to face the mucus barrier. Under physiological conditions the virus particles are transported back to the throat where they are either swallowed or expelled out by coughing. The first druggable target is the integrity and antiviral defense of the mucus with its mucins constituents. Here only a few attempts have been made to block or alleviate damages of the mucus producing epithelia in the airways. Recently, a study appeared that intended to develop interface cultures of the proximal airways to prevent infection with SARS-CoV-2 and the subsequent epithelial cell-autonomous proinflammatory responses. One of the first COVID-19 drug candidates was Remdesivir, which proved to strongly suppress viral infection/replication. These compounds target the virus at the level of viral replication, at the level of the viral RNA polymerase. Potential drugs acting on this level are effective in all cells that expose the ACE2 receptor allowing the virus to invade them. However, only at first glance it appears that those compounds are not specific for the airway system 172. In contrast, the effect of polyP on the ACE2 : RBD interaction is unprecedentedly unique.

**Figure 8:**
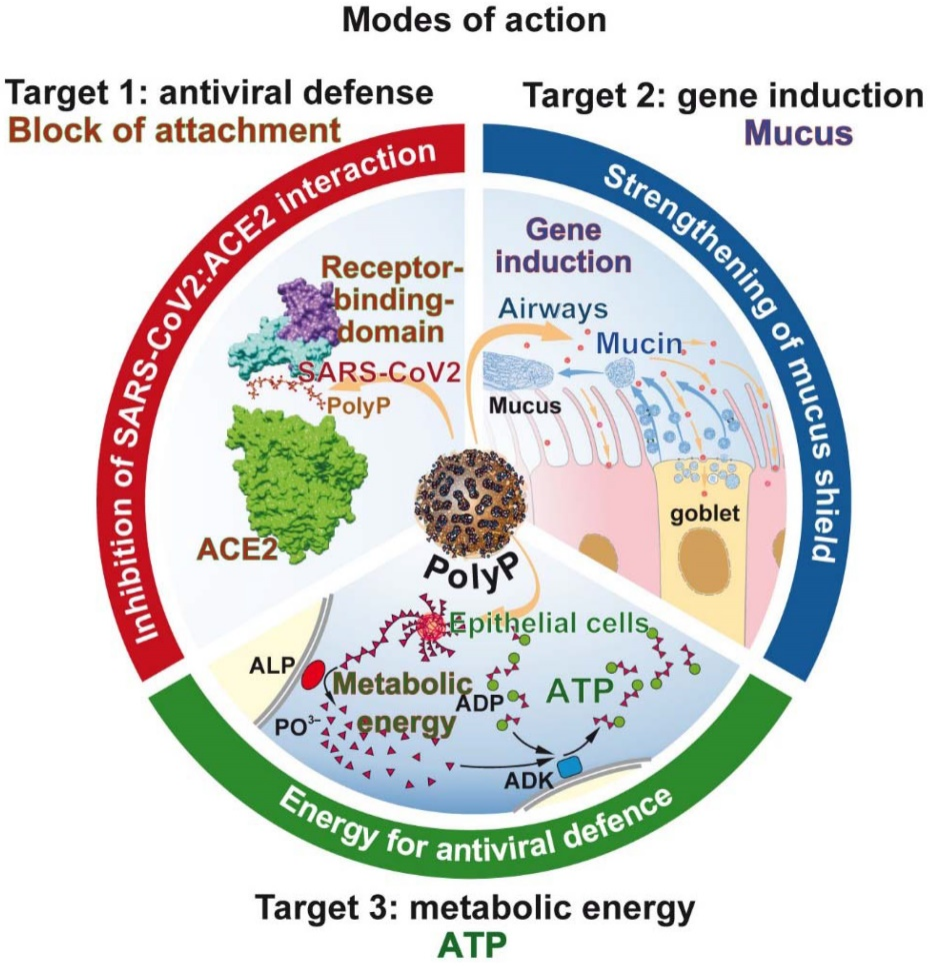
The molecular triad of targets that are induced/strengthened by polyP in the airway system. Target 1: Inhibition of virus attachment to the host cells by masking the binding sites of the virus on the RBD with polyP. Target 2: Induction of genes encoding for mucins, followed by an increased production of the mucin/mucus protective coat around the epithelial cells of the airways. Target 3: Increased synthesis of ATP on the surface of the epithelial cells after exposure to polyP. In turn the cells become resistant to viral infection.

In the center of this review is polyP, with its unique property to act not only as a regeneratively active biomaterial 144, but also as a promising physiological compound preventing SARS-CoV-2 infection. The mode of action of polyP is threefold (Figure 8). It is unusual that a drug has three distinct modes of action.

### Target 1 of polyP: Masking and blocking of SARS-CoV-2

In the center of any antiviral drug development is the screening for specific neutralizing/inhibiting compounds. Since it is difficult to run the RNA polymerizing synthesis process in vitro due to the involvement of membrane components, a large-scale application is not straightforward. Other specific target sites are required. A distinct site of attack is at the interaction of the viral spike RBD protein with the corresponding ACE2 receptor on the target cells. This interaction is specific and involves the unique cationic groove exposed on the RBD surface. From this cationic groove electrostatic forces originate that initiate and maintain a strong interaction to the anionic polymer polyP. As outlined above, the cationic groove is so highly conserved that even the newly, until now, emerging mutants of SARS-CoV-2 did show alterations or changes within the cationic region of the groove.

### Target 2 of polyP: Reinforcement of the mucus barrier

In order to assess the potential of a potential anti-COVID-19 drug to reinforce the protective barrier of the mucus, epithelial lung cells like the A549 cell line 113,114 or BEAS-2B human lung cells, a human non-tumorigenic epithelial cell line 173 can be used that allow a definition of the effects of the compound on the level of mucin gene expression and mucin organization. Both cell types are positive for the viral ACE2 receptor. In this review it is stressed that polyP undergoes coacervation, the formation of a gel-like phase which readily forms a tight layer around the mucin scaffold (Figure 7.I.D). By varying the concentration of the added polyP, the bulkiness AND the extent of interaction with the mucin is controllable. The advantage of the coacervate layer is its distinct modulatory plasticity, pronounced biocompatibility and high accessibility to hydrolytic enzymes, like ALP.

Already earlier it has been demonstrated that polyP comprises inducing activities on the steady-state-expression of structural and functional extracellular molecules like collagen 174 or enzymes that are involved in remodeling processes in the extracellular matrix like matrix metalloproteinases which govern the breakdown of extracellular fibers during normal physiological processes 175. Using A549 cells it is shown that polyP enhances the expression of both MUC1 and MUC5AC both on the gene expression level and on the protein level. It is important to note that the expression of mucin mRNA and protein alone does not indicate an antiviral protection by this family of glycoproteins. To a similar extent the state of glycosylation and the composition of the carbohydrate units are important 176. Similar to polyP, mucins are also polyanionic molecules with the potential to interact with the SARS-CoV-2 S RBD protein.

### Target 3 of polyP: Increasing the metabolic energy state of the system

The special feature of polyP is the property of the polymer to deliver metabolic energy during its biological gene-inducing function. The production of mucins is an energy-requiring process. In the extra-epithelial zone, polyP is subject to degradation via the present ALP with the simultaneous release of Gibbs free energy, which is conserved in ADP/ATP. These energy-rich nucleotides are available for anabolic processes, like here for mucin production 168.

In addition, the synthesis of new virions in the target cell requires of huge amount of energy 177,178. Among the pathways involved are also those of the synthesis of the nucleoside triphosphate substrates for the RNA synthesis in SARS-CoV-2. In this context, a new pathway, an antiviral defense reaction of the cells, has been highlighted, which is induced in the frame of the innate immunity reaction. This pathway involves the interferon-stimulated molecule viperin, a radical S-adenosyl-L-methionine enzyme which synthesizes the chain terminator 3′-deoxy-3′,4′-didehydro-CTP, an inhibitor of the viral RNA dependent RNA polymerase 179. Also this defense mechanism requires the formation of energy-consuming nucleotides as precursors. It must be stressed that after infection of cells, viruses cause a rewiring of the energy balance in order to promote their own replication 180.

The available data indicate that polyP boosts the ATP production in A549 epithelial cells derived from the airways 47. This finding could suggest that cells exposed to polyP produce more ATP and become more resistant to viral infection 181.

### PolyP: The triad of distinct interactions

The advantage of polyP is that it has the potential to be used as a physiological, effective masking and strongly inhibitory polymer against a SARS-CoV-2 infection. The three ways of the specific action of polyP are summarized in Figure 7. (i.) The inhibitory binding of polyP to the RBD of the viral S-protein is tight and pronounced. The inhibition occurs at physiological concentrations, which allows the expectation that with this polymer a re-balancing of an effective mucus barrier appears most likely. This potential for polyP to act as a physiological modulator of mucin function and a potent blocker of the RBD:ACE2 interaction is unprecedented. (ii.) PolyP induces mucins, which are required to strengthen the physical barrier against the virus. (iii.) The polymer delivers metabolic energy for the cells to defend against the virus.

Perhaps in the future other members of the innate protection system like interferon, which inactivates a subsequent step of the viral life cycle after virus attachment, turn out to be similarly effective. Usually interferon-β becomes upregulated after virus infection 182 resulting in the degradation of viral nucleic acids. However, SARS-CoV-2 infection causes a suppression of interferon gene induction and subsequent protein synthesis 183. This effect has even been confirmed in vivo, in patients 184. Very recently, it has been reported that patients treated with inhaled nebulized interferon β-1a showed a significant improvement and rapid recovery from the disease and the symptoms 185.

### PolyP: Level of the polymer during pathogenesis

Unfortunately, polyP is difficult to determine in the circulating blood 186. In turn, other signs, biomarkers can be selected which are correlated with the disease. As mentioned above, the major site of cellular polyP synthesis in vivo are the blood platelets. Very often SARS-CoV-2 patients show the clinical picture of thrombocytopenia 187. This symptom is caused by an increased platelet consumption, which is only partially compensated by an increased platelet production 188. This disequilibrium is most likely caused by an activation of the platelets whose dense granules are filled with polyP 189. The platelets become activated during the association with the viral S-protein 190. This activation is paralleled by a monocyte-platelet aggregation, which is a robust biomarker not only for platelet activity but also for the presence of inflammatory monocytes. Therefore, based on these data a substitution therapy with short-size Na-polyP, around 50 P_i_ units, appears to be advisable. A preliminary study with blood from human donors did not show an impairment of blood clotting even at the high concentration of 50 µg mL^-1^ (Schepler, unpublished).

Due to the abnormally low levels of blood platelets in patients with the clinical picture of thrombocytopenia 187 the German Robert Koch Institute has qualified this alteration as a primary biomarker for COVID-19 191.

### PolyP: Treatment of the formulation for the clinical study

For sterilization either autoclaving (80°C, 1 bar; 20 min) or a filtration procedure through injection filters (pore size 200 nm) was used. After treatment the filtrate was analyzed by dynamic light scattering or by gel filtration. Using the scattering technique, particles were detected not only in the starting material, but also in the filtrate after passing through the 200 nm filters. The size of the NP in the starting suspension with ~80 to 100 nm did not change after ultrafiltration. In addition to this peak particles of a size around 230 nm appear which we attribute to aggregates/coacervate particles. This finding hints that during incubation of the particles in the buffer system the 80-100 nm particles undergo the formation of larger complexes, perhaps driven by coacervation. In addition, this finding also indicates that the consistency of the particles is not solid but highly flexible. In contrast, no particles could be seen in the autoclaved fraction.

In order to verify if in the autoclaved fraction polyP is still present the fractions were subjected to 7 M urea/16.5% polyacrylamide gel electrophoresis and staining with *o*-toluidine blue 113. Fractions of 30 µg were analyzed per slot. After running the gel was stained with *o*-toluidine blue, a stain that interacts with polyP metachromatically 192. After staining of the gel it becomes striking that no polyP was detectable.

## Potential application route of polyphosphate for the treatment of COVID-19 patients

COVID-19 is a respiratory disease: SARS-CoV-2 enters ACE2-positive cells after passing the airway system. Therefore, the preferable application route, especially if it is desired to block the binding of the virus to the airway epithelial cells is to administer the compound as a spray to the nasopharyngeal region. Such an application route has been successfully used also for a vaccine or for asthma therapy 193.

For a potential inhalation/spray/aerosol treatment of humans the polyP must be applied in a stable and a practicable formulation. In the initial study with this polymer a formulation consisting of polyP incorporated in silica nanoparticles (NP) has been successfully applied 113. In order to accelerate the coacervate formation the application of NP prepared from CaCl_2_ and Na-polyP, as Ca-polyP-NP, could be favorable. These particles are amorphous, implying that they are highly biocompatible and prone to metabolic transformation. They are fabricated biomimetically, meaning they contain polyP with an excess of Ca^2+^. Application of these Ca-polyP-NP together with Na-polyP in a spray formulation results in the immediate formation of a coacervate after coming into contact with proteins like mucin in the airways. After coacervate formation, also the polyP present in the particles is ready to be enzymatically hydrolyzed by ALP.

In such a spray formulation, a mixture of Ca-polyP-NP together with soluble Na-polyP is desirable. The Ca-polyP-NP particles are stable, but form a coacervate after protein contact and rapidly release the soluble polymer 54. Of course the concentration of the soluble Na-polyP should exceed the concentration of the particulate fraction since the soluble polyP is required for enveloping the virus particles. A NP size of around 100 nm has been selected in order to compete with the size of SARS-CoV-2 during the endocytotic uptake of the virus. The dimensions of the NP were assessed by dynamic light scattering (Zetasizer Nano ZS90; Malvern Instruments; Malvern; UK) as described 137. A size of *~*100 nm has been chosen for the Ca-polyP NP that are added to the soluble Na-polyP at neutral pH. A commercial FDA-approved buffer system is used.

**Figure 9:**
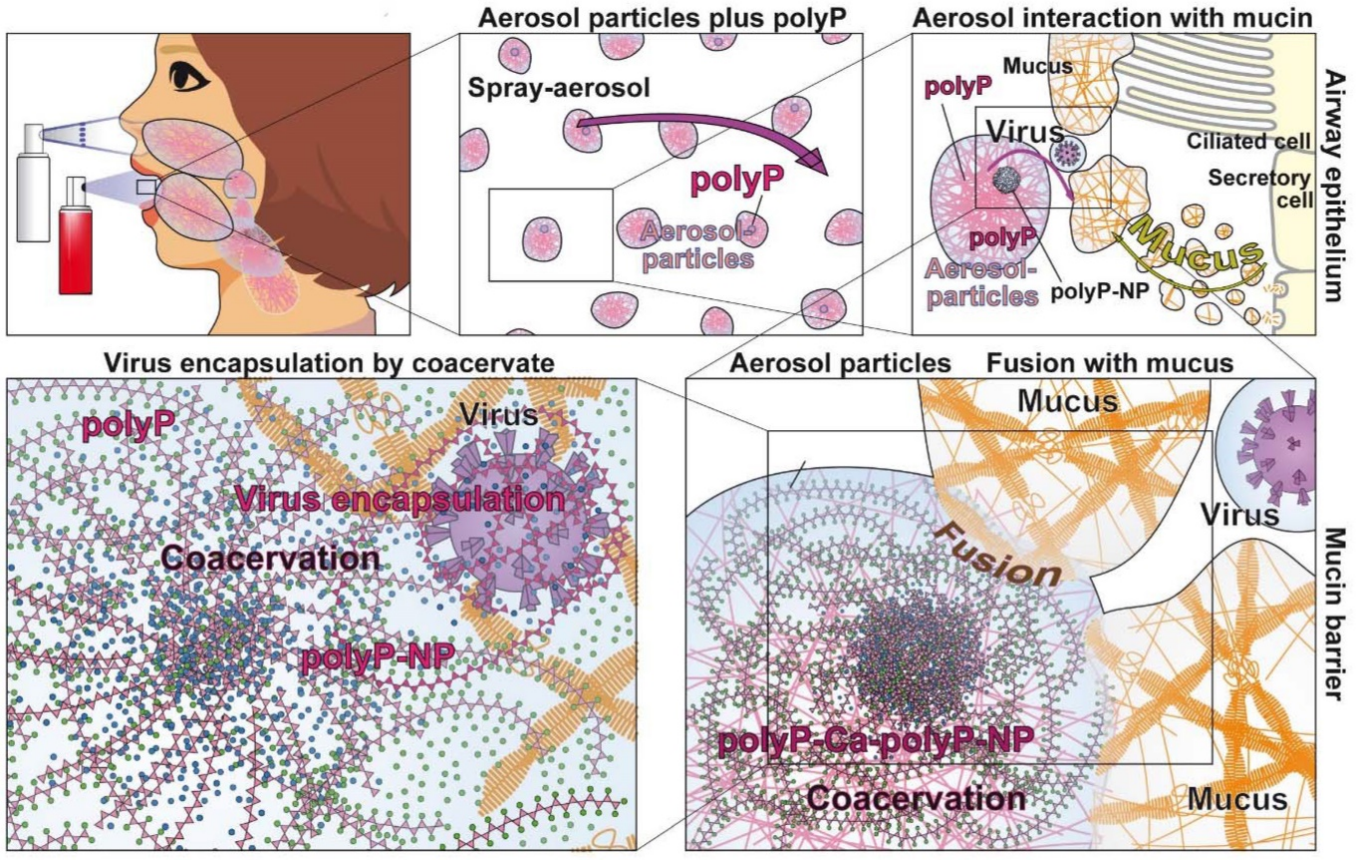
Schematic outline for the application of the polyP formulation for an inhaler which forms under pressure aerosol particles. The polymer, suspended in a pH neutral and isotonic solution can be applied both via the nose or the mouth route. After reaching the mucus-rich epithelia polyP undergoes coacervation a process during which virus particles can be encapsulated.

In the planned study the polyP is dissolved in a buffered solution, with a pH of ~7, matching the pH in the airway surface liquid both in control groups and in patients with airway diseases, like in cystic fibrosis 22. The aqueous polymer solution is dissolved in an isotonic solution and is planned to be administered to the lung by aerosol inhalation. For inhalation different kinds of devices exist; among them a nebulizer, a pressurized metered dose inhaler, and a dry powder inhaler can be used (Figure 9). The first two inhaler devices appear to be most suitable. Both the use for nasal inhalation and for mouth inhalation to the airway system is approached. It is projected to apply three bursts of ~120 µL each two to three times a day. This would allow a daily administration of ~100 µg of active component. The size of the aerosol particles used to distribute the material in the upper airways is between 800 nm and 3 µm. These dimensions would even allow the particles to reach the capillary-rich alveolar region 21. After reaching the mucus layer the polyP particles in the aerosol will be transformed into the coacervate phase, a process allowing the integration of foreign particles, including viruses. After embedding the virus particles will become decorated with polyP chains which attach to the viruses resulting in masking of their S-proteins and prevention of the attachment of the viral particles to the airway epithelia (Figure 6). The process of coacervation is driven by the mucins and the pH (around 7) as well as by the presence of divalent cations 47. In this physico-chemical environment the coacervation also parallels the expansion process of the polymeric mucins after their secretion.

## Future directions

Animal studies are required prior to an application of polyP in clinical trials. In elegant studies it has been demonstrated that polyP protects human intestinal epithelial cells against H_2_O_2_-induced stress 194. Implant studies with Ca-polyP showed that this material is superior to hydroxyapatite/tricalcium phosphate and morselized cancellous bone 145. This beneficial effect is seen only if the implant material is amorphous, allowing the inorganic material to be remodeled into a subsequent crystalline hydroxyapatite bone deposit. It has been proposed that polyP is a beneficial iron chelator and inhibitor of the Fenton reaction 195. PolyP administered locally accelerates wound healing in mice at a concentration of 100 µM (8 µg mL^-1^) 196. PolyP has been introduced as a food additive for human consumption (EC-Number 236-769-6; E452(iv)) and has been qualified as “no hazards classified” in the EINECS inventory 197. Based on these characteristics it appears to be possible also to apply the polymer in human studies, at systemic concentrations of around 10 µg mL^-1^ which can also be reached in vivo after full platelet activation 124.

At the moment polyP can be placed at the same level like interferons, a further member of the innate immunity system. This family of cytokines has the common function to act as antiviral agents that can modulate function of the immune system. Interferon alfa-2a is a member of the standard cocktail for the treatment of chronic hepatitis 198. At present, polyP has the potential to become a potent protecting and perhaps even curing drug against respiratory diseases, especially against COVID-19. Hopefully the clinical studies will support these expectations.

It has to be highly stressed that - in this moment - it is planned to apply for a potential clinical study the short-chain polyP with a chain length of 40 phosphate residues. This choice is based on the finding that - in contrast to the long-chain polyP with a chain length of (>300-1000 P_i_) which might disturb multiple macrophage functions - none of those adverse effects are known for the short-chain polyP. In addition, the short-chain polyP (chain length of ~40 P_i_ units) was found not affect blood clotting extensively, in contrast to the long-chain polyP fraction 140,141. Interestingly, a recent study appeared that even average-chain length of polyP (120 P_i_ units) impaired viral replication 199 and by this confirmed our data on the anti-viral activity of polyP (40 P_i_ units) strongly 113, 114. This group even proposed 199, as reported also in the present review and even before 114, the application of polyP in the form of a nebulized formula for an oropharyngeal delivery to prevent the SARS-CoV-2 infections. What remains open for an efficient application of polyP in patients is the lack of an information on the half-life of the polymer in the mucus. It appears that the duration of polyP is not too short, since the activity of the degrading/hydrolyzing ALP is relatively low 153. However, until now no complications have been identified in in vivo studies 144, 145, using a physiological amorphous nanoparticles formulation introduced by us 146. In a recent study it had been highlighted that polyP acts in vivo as an iron chelator and Fenton inhibitor 195 and blocks by this toxic reactive oxygen species formation during iron stress.

## Abbreviations

ACE2: angiotensin-converting enzyme 2
ADK: adenylate kinase
ALP: alkaline phosphatase
Arg: arginine
Aa: amino acid
COVID-19: coronavirus disease 2019
His: histidine
HIV: human immunodeficiency virus
HSP: heat shock protein
Kb: kilobase
K_D_: dissociation constant
Lys: lysine
N: asparagine
NP: nanoparticles
PO_4_^3-^: phosphate unit
polyP: polyphosphate
R_0_: basic reproductive rate
RBD: receptor binding domain
SARS: severe acute respiratory syndrome
SARS-CoV-2: respiratory syndrome coronavirus 2
Vtc: vacuolar transporter chaperone complex
Y: tyrosine
ΔG°: Gibbs free energy
ζ: zeta potential
